# Plant-Unique *cis*/*trans* Isomerism of Long-Chain Base Unsaturation is Selectively Required for Aluminum Tolerance Resulting from Glucosylceramide-Dependent Plasma Membrane Fluidity

**DOI:** 10.3390/plants9010019

**Published:** 2019-12-23

**Authors:** Masaya Sato, Minoru Nagano, Song Jin, Atsuko Miyagi, Masatoshi Yamaguchi, Maki Kawai-Yamada, Toshiki Ishikawa

**Affiliations:** 1 Graduate School of Science and Engineering, Saitama University, Saitama 338-8570, Japan; m.sato.464@ms.saitama-u.ac.jp (M.S.); kinsyo2007@hotmail.com (S.J.); miyagi@mail.saitama-u.ac.jp (A.M.); yamagu@mail.saitama-u.ac.jp (M.Y.); mkawai@mail.saitama-u.ac.jp (M.K.-Y.); 2 Graduate School of Life Sciences, Ritsumeikan University, Kusatsu, Shiga 525-8577, Japan; mnagano@fc.ritsumei.ac.jp

**Keywords:** rice, glucosylceramide, *cis*/*trans* unsaturation, membrane fluidity, Al^3+^ tolerance

## Abstract

*Cis*/*trans* isomerism of the Δ8 unsaturation of long-chain base (LCB) is found only in plant sphingolipids. This unique geometry is generated by sphingolipid LCB Δ8 desaturase SLD which produces both isomers at various ratios, resulting in diverse *cis*/*trans* ratios in plants. However, the biological significance of this isomeric diversity remains controversial. Here, we show that the plant-specific *cis* unsaturation of LCB selectively contributes to glucosylceramide (GlcCer)-dependent tolerance to aluminum toxicity. We established three transgenic rice lines with altered LCB unsaturation profiles. Overexpression of SLD from rice (OsSLD-OX), which preferentially exhibits *cis*-activity, or Arabidopsis (AtSLD-OX), showing preference for *trans*-activity, facilitated Δ8 unsaturation in different manners: a slight increase of *cis*-unsaturated glycosylinositolphosphoceramide (GIPC) in OsSLD-OX, and a drastic increase of *trans*-unsaturated GlcCer and GIPC in AtSLD-OX. Disruption of LCB Δ4 desaturase (*des*) significantly decreased the content of GlcCer. Fluorescence imaging analysis revealed that OsSLD-OX and AtSLD-OX showed increased plasma membrane fluidity, whereas *des* had less fluidity, demonstrating that the isomers universally contributed to increasing membrane fluidity. However, the results of a hydroponic assay showed decreased aluminum tolerance in AtSLD-OX and *des* compared to OsSLD-OX and the control plants, which did not correlate with membrane fluidity. These results suggest that *cis*-unsaturated GlcCer, not GIPC, selectively serves to maintain the membrane fluidity specifically associated with aluminum tolerance.

## 1. Introduction

Sphingolipids are composed of the unique hydrophobic moiety ceramide, the fatty acyl derivative of long-chain base (LCB), and are major components of the plasma membrane in all eukaryotes [1,2]. In plants, the ceramide moiety is subject to multiple chemical modifications, such as unsaturation and hydroxylation, and combinations of these modifications give rise to an enormous number of molecular species of plant sphingolipids [3]. Sphingolipids are involved in signal transduction through the formation of membrane nanodomains [4] and are implicated in various plant physiological functions such as defense responses to chilling [5,6,7,8], drought [9], salt [10,11], pathogens [12,13], reactive oxygen species [14], hypoxia [15], and Al^3+^ [16]. However, little is known regarding how the plant-specific diversity of sphingolipid structures contributes to these physiological roles.

Dihydroceramide desaturase (DES) and sphingoid LCB desaturase (SLD) are conserved enzymes that introduce the double bond at the Δ4 and Δ8 positions of LCB, respectively ([17,18]; Figure S1). DES converts sphinganine (d18:0) to sphingosine (4-*trans*-sphingenine, d18:1^4^), a common LCB species in most eukaryotes. SLD is found widely in plants, fungi, diatoms, and some invertebrates, but appears to have a unique enzymatic property in plants: SLDs from other organisms produce only the *trans* isomer of the double bond, whereas plant SLDs also have *cis*-unsaturating activity and generate both isomers [17,19,20,21,22]. In addition, the *cis*/*trans* ratios of SLD products are highly diverse and apparently determine the isomeric compositions of the final product glycosphingolipids in each plant [5,23]. For example, SLDs from Arabidopsis and many dicots predominantly generate the *trans* bond rather than the *cis*, while enzymes from monocots preferentially generate the *cis* configuration, roughly consistent with the isomer ratios in those plants [5,23,24,25].

In general, unsaturated bonds in lipids interfere with lipid inter- and intra-molecular hydrophobic interactions, resulting in more fluidic lipid bilayers, in which the *cis* configuration provides a much larger effect than *trans*. Indeed, it was previously demonstrated that the effect of isomerization on the fluidity of artificial membranes follows the order *cis* > *trans* > saturated fatty acids in glycerolipids [26]. Membrane fluidity has been well studied in association with chilling tolerance in plants, and several studies suggest an additional role in aluminum tolerance. One toxic action of Al^3+^ is its binding to the negative charges of phospholipids on the surface of the plasma membrane, disturbing membrane fluidity and function [27,28,29]. The unsaturation rates of fatty acids are elevated under aluminum stress in Al-tolerant cultivars of rice [30]. Pejchar et al. [31] reported that non-specific phospholipase 4 is closely associated with early and long-term responses to aluminum stress in Arabidopsis. Although there have been no studies of the physical interactions between Al^3+^ and sphingolipids containing a phospho-group, Ryan and colleagues [16] reported that LCB Δ8 unsaturation and its *cis*/*trans* isomer ratio are associated with aluminum tolerance in Arabidopsis. In contrast, yeast ectopic expression studies provided ambiguous results regarding isomer-specific function. Yeast strains expressing plant SLDs show aluminum-resistant phenotypes but there is no apparent correlation between the degree of tolerance and the *cis*/*trans* ratio [16,23,32] and thus the functional distinction between LCB *cis*/*trans* isomers remains controversial. In addition, it is unknown how sphingolipid classes, with each class comprising various head groups and ceramide backbones, are associated with aluminum toxicity and tolerance due to LCB unsaturation. Plants contain two classes of glycosphingolipids, glucosylceramide (GlcCer) and glycosylinositolphosphoceramide (GIPC), as major components of the plasma membrane. The GIPC head contains a negatively charged phospho-group and a glucuronic acid residue [3,33,34], both putative targets for Al^3+^ binding. In addition, the two glycosphingolipid classes have distinct ceramide moieties in the LCB Δ8 structure. GlcCer selectively incorporates Δ8 unsaturated LCBs at a higher *cis*/*trans* ratio, while GIPC prefers Δ8 *trans*-unsaturated or saturated species ([3,25,35]; Figure S1). How are these unique classes associated with aluminum toxicity and tolerance thorough LCB unsaturation and membrane fluidity?

In this study, we focused on the role of *cis* unsaturation of sphingolipids in membrane fluidity-dependent aluminum tolerance. We evaluated the importance of plant-specific LCB structures by choosing rice (*Oryza sativa* L.) as a model plant because of its *cis*-enriched LCB composition in endogenous sphingolipids. Our results obtained by gain- and loss-of-function experiments provide evidence that the *cis* configuration of LCB Δ8 unsaturation is required for aluminum tolerance by maintaining membrane fluidity in a lipid class-dependent manner.

## 2. Results

### 2.1. Different Isomer Productivity of Arabidopsis and Rice SLDs

**Figure 1:**
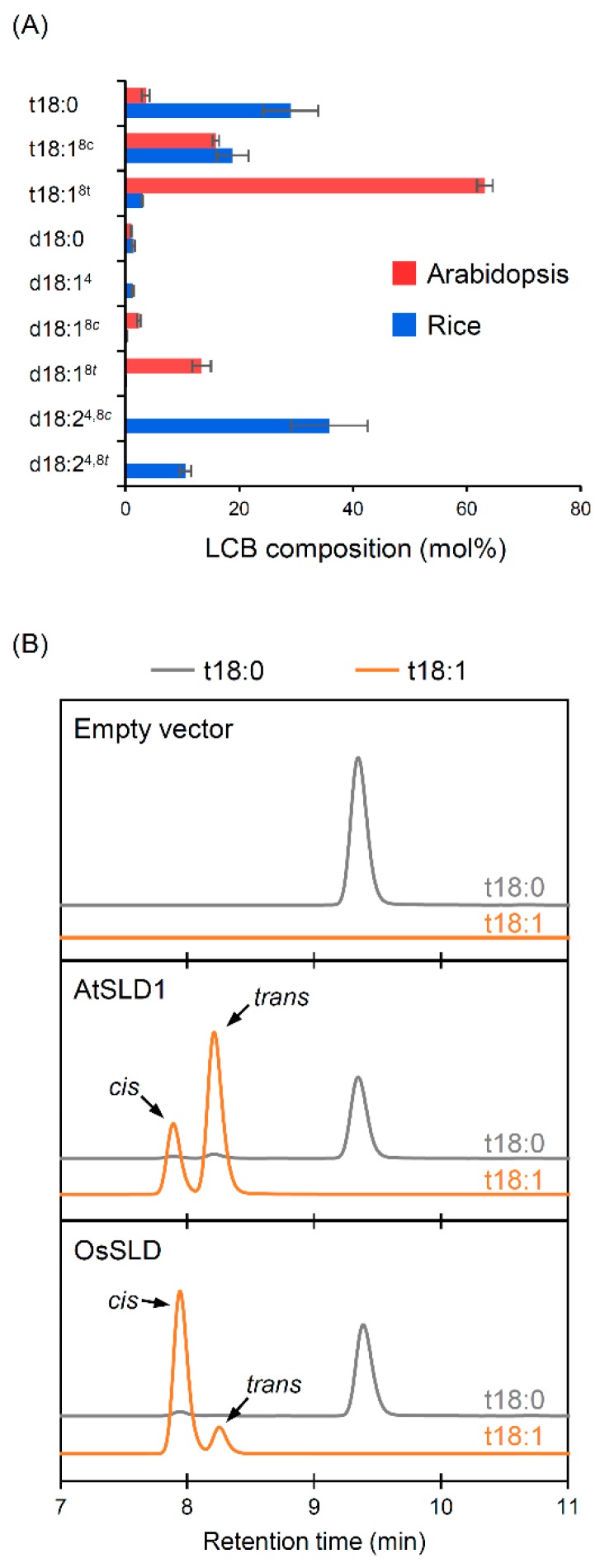
Different isomerism of long-chain base (LCB) Δ8 unsaturation in rice and Arabidopsis. (**A**) Total LCB profiles in vegetative leaves of Arabidopsis and rice. Data are means ± SD (*n* = 4). (**B**) LC-MS/MS chromatograms of t18 LCBs in yeast expressing AtSLD1 or OsSLD. 4-nitro-7-benzofurazan (NBD-F) derivatives of t18:0 and t18:1 were monitored by *m*/*z* 479.4 > 193.1 and 477.4 > 193.1, respectively.

The *cis*/*trans* ratio of LCB Δ8 unsaturation differs greatly among plant species [5,24,35]. Total LCB profiles in the leaves of Arabidopsis and rice are shown in Figure 1A. Major Δ8 unsaturated species of trihydroxy (t18) and dihydroxy LCBs (d18) were commonly detected (t18:1^8^ and d18:1^8^ in Arabidopsis; t18:1^8^ and d18:2^4,8^ in rice), but the *cis*/*trans* ratios were opposite, i.e., *trans*-predominant in Arabidopsis and *cis*-predominant in rice. The results of a yeast ectopic expression assay confirmed that the plant species-specific isomerism of LCB Δ8 unsaturation is due to characteristics of the enzymes from each plant: AtSLD1 from Arabidopsis and OsSLD from rice showed desaturase activity to convert t18:0 to t18:1 at the typical *cis*/*trans* ratio consistent with that observed in each plant **(**Figure 1B).

### 2.2. Genetic Modification of the LCB Composition in Rice

Our aim was to analyze the biological importance of the plant-specific Δ8 *cis* unsaturation of LCB and thus we chose rice as a *cis*-predominant plant for genetic experiments. We first attempted disruption of *OsSLD* by CRISPR/Cas9-mediated genome editing. However, no homozygous mutant was obtained, even though partial nucleotide mutation occurred in the vegetative tissues of transgenic plants harboring the CRISPR/Cas9 cassette (see details in Figure S2). RNAi was also tried but sufficiently knock-downed plants could not be isolated. We concluded that functional deficiency of *OsSLD* causes lethality in rice.

**Figure 2:**
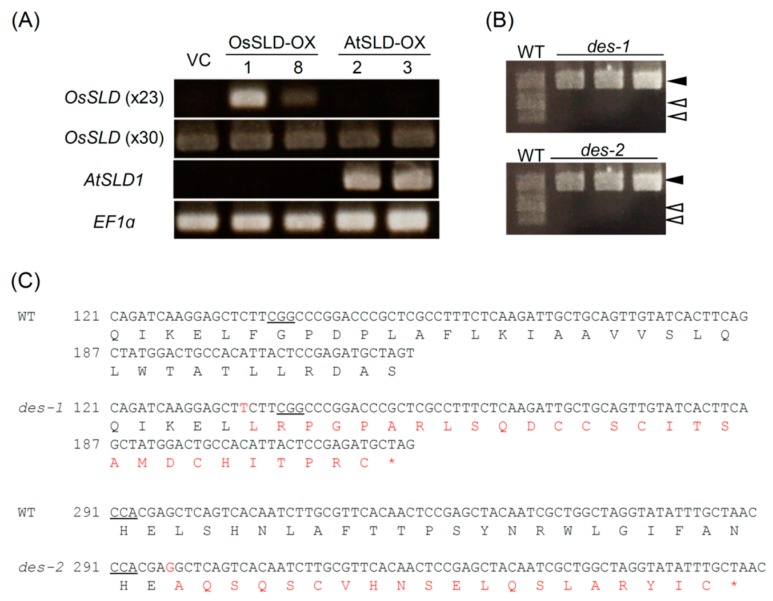
Characterization of transgenic rice plants. (**A**) RT-PCR analysis of *OsSLD* and *AtSLD1* in OsSLD-OX and AtSLD-OX lines. *EF1α* was used as a reference gene. The *OsSLD* amplicon was detected in only OsSLD-OX plants at 23 cycles of PCR but in all lines at 30 cycles. *AtSLD1* was never amplified except for AtSLD-OX. VC—vector control. (**B**) Restriction digestion of genomic PCR products including CRISPR/Cas9 target sites for *OsDES* in *des-1* and *des-2* plants. Closed and open arrowheads indicate non-digested PCR products and cleaved fragments, respectively. (**C**) Nucleotide and deduced amino acid sequences of *OsDES* cDNA. Red-colored letters show altered sequences in the mutants. An asterisk shows the stop codon. PAM sequences (NGG) are underlined.

Our failure to isolate rice *sld* mutants prompted us to prepare transgenic rice using different approaches. Enzymatic conversion of the double bond from *cis* to *trans* was enabled by overexpressing AtSLD1 in rice under the strong maize *Ubi-1* promoter (AtSLD-OX). An OsSLD overexpressor (OsSLD-OX) was also established to enhance t18:1^8*c*^ production from t18:0 abundant in wild-type rice (Figure 1A). Transgenic plants with higher expression levels of the transgenes were isolated (Figure 2A) and used for further analyses.

In addition, we generated disruptants of *OsDES* encoding LCB Δ4 desaturase. In *Pichia pastoris*, SLD necessitates Δ4 unsaturated LCB (d18:1^4t^) as the substrate and the *des* mutant is unable to produce Δ8 unsaturated sphingolipids [36]. Similar to *P*. *pastoris*, Δ4 and Δ8 di-unsaturated LCB (d18:2^4,8^) is the most abundant Δ8 unsaturated LCB in rice and thus we anticipated a negative effect of *DES* mutation on Δ8 unsaturation in rice. Unlike *OsSLD*, we obtained homozygous mutants of the *OsDES* gene using a similar CRISPR/Cas9 system (Figure 2B; Figure S3). We isolated two independent mutant lines generated using different target sequences (*des-1* and *des-2*). Sequencing analysis confirmed that both contained a one nucleotide insertion within the coding sequence, likely causing null deficiency of DES activity due to a frameshift mutation near the N-terminal end of the polypeptide (Figure 2C).

### 2.3. Alteration of LCB Profiles in Transgenic Rice

**Figure 3:**
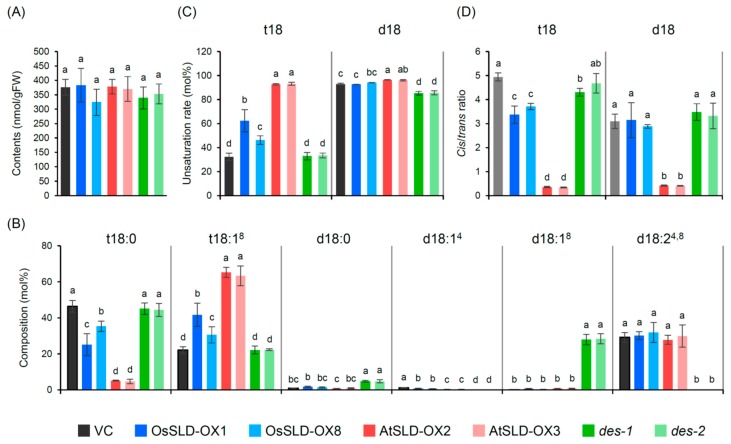
LCB profiling of the transgenic rice plants. LCBs were liberated from root tissues by direct strong alkaline hydrolysis and analyzed by LC-MS/MS. (**A**) Total LCB contents. (**B**) Composition of LCB species. (**C**) Unsaturation rate (mol% of Δ8 unsaturated species each of t18 and d18 LCB). (**D**) *Cis*/*trans* ratio of Δ8 unsaturation. Data are means ± SDs (*n* = 4). Different letters indicate statistical significance (Tukey’s HSD, *P* < 0.05).

In association with aluminum tolerance in roots, we analyzed total LCB profiles in the transgenic rice plants by direct hydrolysis of root tissues. All the transgenic plants and the vector control (VC) showed comparable levels of total LCB but different LCB compositions according to the genetic modifications (Figure 3). OsSLD-OX plants showed an ~20% decrease of t18:0 and an equivalent increase of t18:1, indicating that t18:0 was partially unsaturated (Figure 3B,C). The *cis*/*trans* isomer ratio of t18:1 in OsSLD-OX slightly decreased compared to that in VC but remained high (Figure 3D). The amount, unsaturation rate and *cis*/*trans* ratio of d18 LCBs were unaffected by OsSLD overexpression, showing that overexpression of OsSLD only slightly affected t18 unsaturation in rice. In contrast, AtSLD-OX had drastic effects on LCB composition: t18:0 was almost completely converted to t18:1 and its *cis*/*trans* ratio was ~0.3, similar to that of the product of AtSLD1 expressed in yeast cells. The amounts of d18 LCBs were not affected but the *cis*/*trans* ratio of the d18:2^4,8^ species was also ~0.3, indicating that ectopically expressed AtSLD1 much more effectively desaturates endogenous substrates compared to OsSLD.

We did not detect LCB species containing Δ4 unsaturation in the *des* mutants, demonstrating complete disruption of enzyme activity. However, d18:1^8^ in *des* reached a level similar to that of d18:2^4,8^ in the control plants, indicating that endogenous OsSLD could accept both d18:1^4^ and d18:0 as substrate. This finding indicates that OsSLD has substrate specificity similar to AtSLD1 [5] rather than to *P*. *pastoris* SLD.

### 2.4. Characterization of the Sphingolipidomic Profile of Transgenic Rice Root Tissue

**Figure 4:**
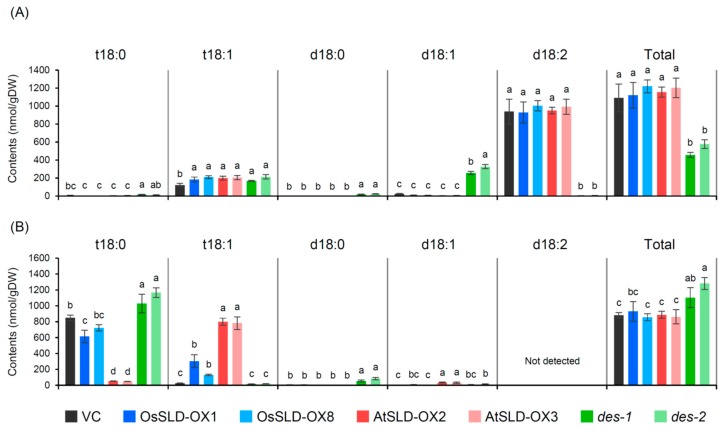
Sphingolipidomic characterization of transgenic rice plants. LCB profiles and total contents of (**A**) glucosylceramide (GlcCer) and (**B**) glycosylinositolphosphoceramide (GIPC). Data are means ± SDs (*n* = 3–4). Different letters indicate statistical significance (Tukey’s HSD, *P* < 0.05). VC, vector control.

We studied how the above-described alterations in LCB composition impacted complex glycosphingolipids by performing sphingolipidomic analysis of the root tissues of transgenic rice. Figure 4 shows profiles of the two major glycosphingolipid classes, GlcCer (A) and GIPC (B). Consistent with previous reports, both classes displayed distinct LCB compositions in their ceramide moieties: GlcCer primarily contained Δ8-unsaturated LCBs but GIPC was mainly composed of saturated t18:0 (Figure 4; [25]). In OsSLD-OX and AtSLD-OX, t18:0 decreased and t18:1 increased in GIPC, whereas t18:1-containing GlcCer was only slightly elevated. These results showed that accumulated t18:1 LCB in these plants was incorporated predominantly into GIPC rather than into GlcCer, regardless of the *cis*/*trans* ratio. Di-hydroxy LCB is mainly d18:2 and was restricted in GlcCer but not in GIPC, indicating that the drastically altered *cis*/*trans* ratio of d18 LCB in AtSLD-OX (Figure 3D) occurred in GlcCer. Unexpectedly, d18:1 accumulated but its level was much lower than the loss of d18:2 in the *des* mutants, inconsistent with their LCB profiles shown in Figure 3. Significant levels of d18:1 were not detected, even when MS/MS-targeted monitoring was further extended to include species of free ceramide, free LCB, and unusual GlcCer and GIPC species containing non-hydroxylated fatty acids and/or non-standard sugar head groups (data not shown). Based on these data, although it is possible that d18:1^8^ is incorporated into unknown ceramide derivatives, the level of GlcCer with Δ8 unsaturation drastically decreased in *des* mutants to ~40% of that in control plants.

### 2.5. Imaging Analysis of the Fluidity of the Plasma Membrane

**Figure 5:**
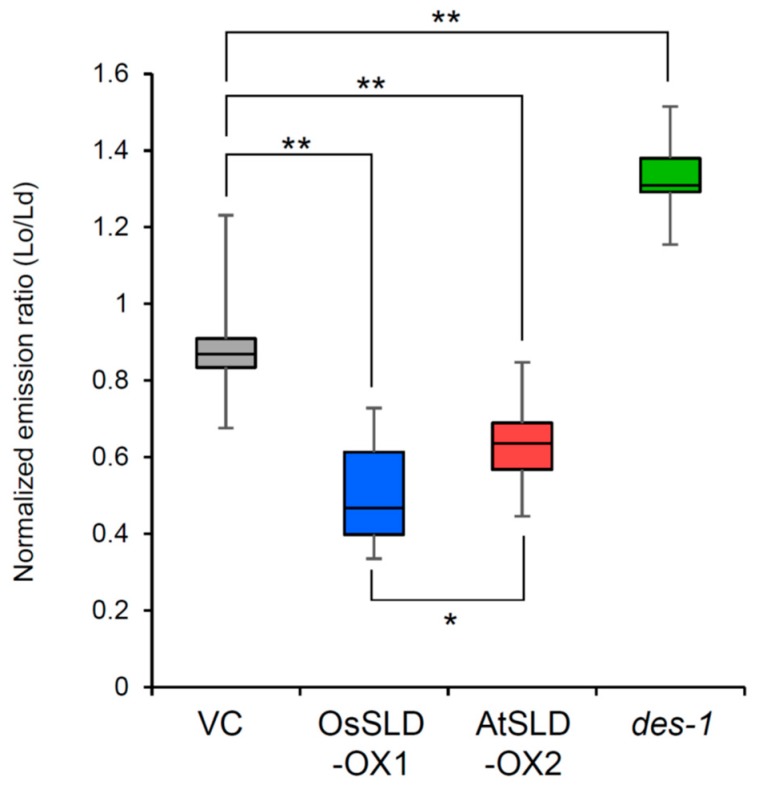
Membrane order in transgenic rice plants. Plasma membrane fluidity was analyzed using normalized liquid-ordered (Lo) and liquid-disordered (Ld) emission using di-4-ANEPPDHQ. Data are presented as box plots, with the boxes representing the interquartile range. Lines extend from the box to the highest and lowest values. The median value is represented by the thick line across each box. Statistical significance was assessed by one-way ANOVA (*n* = 11–14, * *P* < 0.01, ** *P* < 0.001).

We assessed the fluidity of the plasma membrane of the root epidermis in the transgenic lines by visualizing the liquid-ordered (Lo) and liquid-disordered (Ld) membrane phases in vivo using the fluorescent indicator di-4-ANEPPDHQ [13,37]. Confocal laser scanning microscopy showed that the Lo/Ld ratio was significantly reduced in OsSLD-OX and AtSLD-OX compared to that in VC, demonstrating that both isomers of LCB unsaturation contribute to an increase in plasma membrane fluidity (Figure 5; Figure S4). OsSLD-OX showed the highest fluidity, despite a slight increase of Δ8 unsaturation only in GIPC. This finding indicates that t18:0-containing GIPC forms a Lo phase in the plasma membrane and *cis* unsaturation of the t18:0-GIPC effectively changed the membranes to the Ld phase. AtSLD-OX also showed increased membrane fluidity but less than OsSLD-OX, despite the LCB moieties of most sphingolipids being unsaturated. Given that *cis*-unsaturated GlcCer was mostly converted to *trans* in AtSLD-OX, as shown above, *trans* unsaturation of LCB could moderately increase membrane fluidity but less effectively than *cis* unsaturation. On the other hand, the Lo/Ld ratio in *des* was significantly elevated, indicating decreased plasma membrane fluidity. Compared to the control, this mutant contained less GlcCer with d18:2^4,8^ but a comparable level of GIPC, suggesting that compared to GIPC, *cis*-unsaturated GlcCer tends to form a disordered membrane. These results indicate that LCB unsaturation increases the fluidity of a membrane in a similar manner to the fatty acyl chains of glycerolipids.

### 2.6. Aluminum Tolerance Assay

**Figure 6:**
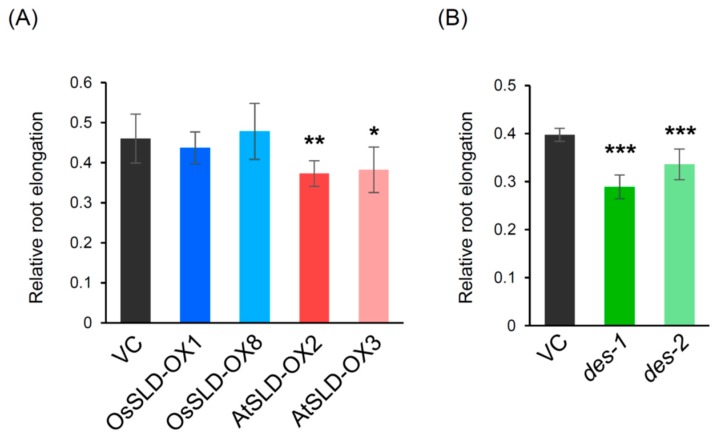
Aluminum tolerance in rice roots. Al^3+^ tolerance in SLD-OX (**A**) and *des* mutant (**B**) was evaluated as relative root elongation (ratio of root length elongated in treated/non-treated conditions). Two typical data obtained independent experiments are shown. Data are means ± SDs (*n* = 8). Asterisks indicate statistical significance between vector control (VC) and each transgenic plant (student’s *t*-test, * *P* < 0.05, ** *P* < 0.01, *** *P* < 0.001).

Aluminum tolerance was assessed by measuring root elongation using a hand-made hydroponic culture system (Figure S5). In the absence of aluminum stress, the transgenic rice plants showed slightly different elongation rates of the primary root in the early development. Thus, we evaluated aluminum tolerance by relative root elongation (ratio of Al^3+^/non-treated). First, we analyzed the SLD-overexpressing plants (Figure 6A). In the control plants root elongation was repressed by 50–60% under the presence of 50 µM Al^3+^, and OsSLD-OX showed comparable tolerance. In contrast, AtSLD-OX showed significant suppression of root elongation compared to VC and OsSLD-OX. Similar experiments using *des* mutants showed that Al^3+^-induced retardation of root elongation was significantly exacerbated in the two mutant lines (Figure 6B). These results indicate that sphingolipids are closely associated with aluminum tolerance in rice, but membrane fluidity (Figure 5) and aluminum tolerance are not correlated in a simple manner. Below we provide a hypothesis describing a sphingolipid class-dependent relationship between membrane fluidity and aluminum toxicity.

## 3. Discussion

The *cis* double bond at the Δ8 position of LCB is a structure unique to plant sphingolipids. Ryan et al. [16] reported elevated tolerance to aluminum stress in Arabidopsis expressing a *cis*-preferring SLD isolated from *Stylosanthes hamata*. However, in a later study, Li et al. [23] analyzed the aluminum tolerance of yeast expressing a wide variety of plant SLDs and observed no correlation between aluminum tolerance and the LCB *cis*/*trans* ratio. Although *cis* unsaturation generally provides higher membrane fluidity and thus enhanced aluminum tolerance, this conclusion in the case of plant sphingolipids remains contentious. In addition, LCB structures are thought to play an important role in the selective distribution of ceramide backbones into the two major classes, GlcCer and GIPC [5]. GIPC contains negative charges in the head group [3,25] that are possible targets of Al^3+^ binding on the surface of the plasma membrane as reported in phospholipids [27], causing aberrant membrane fluidity and function. To our knowledge, however, there is no report addressing the class-specific association of sphingolipids in aluminum tolerance. In this study, we chose rice as a model plant for the in planta evaluation of the biological importance of the plant-specific *cis* isomer by genetic modification. Transgenic rice plants with altered LCB and sphingolipidomic compositions were established and analyzed for membrane fluidity and aluminum tolerance. Below we discuss our findings, focusing on two viewpoints: metabolic impacts of LCB unsaturation on the rice sphingolipidome (*3.1*), and the relationship between sphingolipid structure, membrane fluidity, and aluminum tolerance (*3.2*).

### 3.1. Metabolic Effects of the Genetic Modification of LCB Unsaturation on the Sphingolipidome in Rice

The two major sphingolipid classes in plants, GlcCer and GIPC, are composed of distinct ceramide backbones. GIPC predominantly contains Δ8 *trans*-unsaturated or saturated LCB, whereas GlcCer preferentially contains Δ8 *cis*-unsaturated LCB [5,38]. In rice, most GIPC molecules have saturated t18:0 LCB whereas GlcCer contains unsaturated d18:2 and t18:1 at a high *cis*/*trans* ratio ([25]; Figure 3; Figure 4). These observations suggest that the metabolic distribution of ceramides into the two glycosphingolipid pools depends on structural information in the LCB moiety, e.g., the *cis* and *trans* geometry of LCB unsaturation determines ceramide influx into the GlcCer and GIPC pools, respectively.

We generated strong overexpressors of *OsSLD* and *AtSLD1* in rice (Figure 2). Wild-type rice contains saturated t18:0 as a major LCB (Figure 1) and thus OsSLD overexpression was estimated to result in the accumulation of t18:1 at a high *cis*/*trans* ratio, which would further result in enhanced influx of ceramide into the GlcCer pool. However, t18:1 was only slightly overproduced from its substrate t18:0 in OsSLD-OX, and surprisingly, the unsaturated product appeared to be incorporated into the GIPC pool more than into GlcCer (Figure 4). In contrast, overexpression of AtSLD1 greatly enhanced the Δ8 unsaturation of t18 at a lower *cis*/*trans* ratio and the *trans*-unsaturated LCB was effectively incorporated into GIPC. These results suggest that ceramide distribution into GlcCer and GIPC does not always depend on the LCB Δ8 structure, and LCB *cis* unsaturation itself could be restricted by an unknown regulatory machinery that determines the pool sizes of glycosphingolipids.

In this study, we could not isolate homozygous knock-out mutants of rice for *OsSLD*, indicating that this enzyme is essential in rice. This finding is inconsistent with previous reports regarding other organisms such as Arabidopsis and *Candida albicans*, in which *SLD* null mutants were not lethal despite complete loss of Δ8-unsaturated sphingolipids, although the mutants showed aberrant phenotypes in their hyphal growth and under stressed conditions [5,39]. A common feature of these organisms is to have the *trans* configuration at the Δ8 position of LCB, implying that the loss of *cis*-predominant unsaturation in rice has a much larger effect than the alteration from *trans* to saturated LCBs in other organisms. Further studies using other species showing rice-like *cis*-rich LCB unsaturation are necessary to confirm the essentiality of *cis* unsaturation in such organisms.

Δ4 unsaturation of LCB is a more common structure than Δ8 in eukaryotic organisms [18]. In plants, however, the functions of the responsible enzyme DES have only been studied in Arabidopsis, in which *AtDES* expression and production of the Δ4-unsaturated species are restricted to pollen [40,41]. In addition, AtSLD1 and AtSLD2 can convert d18:0 to d18:1^8^, and the product is incorporated into GlcCer in the pollen of *des* and in most tissues of wild-type plants, leading to no visible phenotype in the *des* mutant of Arabidopsis [41]. In *P. pastoris*, on the other hand, DES deficiency causes a decrease in Δ8 unsaturation and GlcCer biosynthesis [36,40]. Here we showed that rice SLD could desaturate d18:0 to d18:1^8^ in vivo, similar to Arabidopsis SLDs, but the product was not effectively incorporated into GlcCer, resulting in a drastic decrease in GlcCer in *des* mutants (Figure 3 and Figure 4). This result suggests that the metabolic usability of ceramide in the GlcCer pool differs between Arabidopsis and rice. Curiously, the accumulated d18:1 LCB detected in our total LCB analysis was absent from our sphingolipidomic analysis. Since LCB desaturases appear to act on ceramide rather than free base [5,42], this observation implies that the unusual d18:1^8^ LCB-containing ceramide might be metabolized to unknown derivatives. Our findings regardless demonstrate that the Δ4 unsaturation of LCB is required for its influx into the GlcCer pool in rice unlike in Arabidopsis, suggesting that the function of LCB Δ4 unsaturation remains to be addressed in various plant species.

### 3.2. Relationship between LCB Unsaturation, Membrane Fluidity, and Aluminum Tolerance

One mechanism of aluminum toxicity involves Al^3+^ binding to the negative charges of phospholipids on the surface of the plasma membrane, thus disturbing membrane fluidity and function [27,28,29]. In addition, aluminum stress stimulates expression of fatty acyl desaturases, leading to a higher degree of unsaturation of glycerolipids in aluminum-tolerant rice cultivars compared to susceptible cultivars [30]. These observations support that both the surface charges and fluidic states of the plasma membrane are closely associated with tolerance to aluminum toxicity. Plant sphingolipids comprise two classes with distinct head groups and ceramide backbones but it remains poorly understood how these two classes differently contribute to membrane fluidity and aluminum tolerance. In particular, plant sphingolipids contain LCBs with Δ8 *cis*/*trans* isomerism. The *cis* geometry, in general, increases membrane fluidity much more effectively than the *trans* configuration [26,43]. We evaluated the biological importance of the plant-specific *cis* isomer of unsaturated LCB by analyzing the membrane fluidity and aluminum tolerance of transgenic rice plants. Imaging analysis using di-4-ANEPPDHQ showed that the Lo/Ld ratio decreased in OsSLD-OX and AtSLD-OX but increased significantly in *des* (Figure 5), clearly demonstrating that both isomers of LCB Δ8 unsaturation contribute to membrane fluidity, with *cis* having a greater effect than *trans*. This is consistent with the previous report on the desaturation of fatty acyl chains in phosphoglycerolipid membranes [26]. However, aluminum tolerance was not correlated with membrane fluidity: AtSLD-OX and *des*, with decreased and increased Lo/Ld, respectively, showed significantly lower aluminum tolerance compared to the control. Overexpression of AtSLD1 seems to have a complex effect on membrane fluidity through different alterations of LCB unsaturation in GlcCer and GIPC. Our findings suggest that changing most t18:0 to t18:1^8*t*^ increases the fluidic state of GIPC-formed membranes, whereas the *cis*-to-*trans* conversion of d18:2 causes a decrease in GlcCer-based membrane fluidity. Furthermore, *des* mutant rice has a significantly lower level of GlcCer yet shows a similar aluminum-sensitive phenotype, suggesting that the fluidity of GlcCer-formed membranes rather than GIPC membranes is specifically required for tolerance to aluminum toxicity. This idea also explains why OsSLD-OX had unaltered aluminum tolerance despite having the highest membrane fluidity: the increased fluidity in OsSLD-OX was due to the increased unsaturation of GIPC rather than to the presence of GlcCer. Although GIPC may be a direct target of Al^3+^, our results suggest that GlcCer, an apparent non-target of Al^3+^, contributes to aluminum tolerance by maintaining a specific membrane fluidity. GlcCer and GIPC have distinct ceramide structures, suggesting that these two glycosphingolipid classes form distinct phases in the plasma membrane. The structural feature of GIPC, a longer fatty acyl chain and a lower unsaturation rate, has been estimated to form the interdigitated rigid membranes, whereas the shorter chain length and higher unsaturation rate with the cis configuration of GlcCer would interact with more fluidic membrane components [4]. Further studies to elucidate the spatial relationships between the lateral compartmentalization of membrane lipids and the target binding sites of Al^3+^ will help us understand the molecular basis of aluminum toxicity in the plant plasma membrane.

In summary, we here provide experimental evidence of the contribution of LCB unsaturation to membrane fluidity and aluminum tolerance in lipid class- and stereo-specific manners. These findings strongly support the idea that GlcCer-targeting modification using *cis*-type LCB desaturases holds promise as a new tool for improving aluminum tolerance in crop plants.

## 4. Materials and Methods

### 4.1. Plant Growth Conditions

For genotyping and leaf LCB profiling, rice (*Oryza sativa* L. cv. Nipponbare) seeds were germinated in a Petri dish and then transferred to soil. To collect root materials, seeds were sown on a plastic net floating on distilled water. Rice seedlings were grown at 28 °C under a 12 h light (300 µmol/m^2^/s)/12 h dark cycle. Arabidopsis plants were cultivated at 22 °C under continuous light (60 µmol/m^2^/s).

### 4.2. LCB Analysis

Total LCB was liberated from fresh rice tissues by hydrolysis in barium hydroxide/dioxane for 24 h at 110°C and analyzed by LC-MS/MS, followed by derivatization with 4-nitro-7-benzofurazan (NBD-F) according to a published method [44]. The amount of LCB in each sample was normalized using d17:1 (Avanti Polar Lipids) as an internal standard and represented as nmol per g fresh weight (FW).

### 4.3. Yeast Expression Assay

Coding sequences of *AtSLD1* (At3G61580) and *OsSLD* (Os09g0338500) were cloned into pGK423 [45] and introduced into *Saccharomyces cerevisiae* W303 strain. Cells were collected from overnight culture (1 mL) and subjected to LCB analysis as described above.

### 4.4. Transgenic Rice

CRISPR/Cas9 target sequences of *OsSLD* and *OsDES* (Figures S2 and S3) were designed using the CRISPR-P tool [46] and cloned into pZH_gYSA_MMCas9 [47]. Agrobacterium-mediated transformation of rice was performed as described previously [14]. Genomic DNA including the target site was PCR amplified and digested by appropriate restriction enzymes (Figures S1 and S2). The primer sequences used are shown in Supplementary Table S1.

For overexpression, the coding sequences of *AtSLD1* and OsSLD were cloned into pRiceFOX-GateA under the maize *Ubi-1* promoter and introduced into rice calli via *Agrobacterium tumefaciens* strain EHA105. Homozygous transformants (T3–T4 generation) were used for the experiments.

### 4.5. RT-PCR

Total RNA was extracted from leaves of wild-type and transgenic rice using an RNeasy Plant Mini kit (Qiagen, Hilden, Germany). cDNA was prepared using a High Capacity cDNA reverse transcription kit (Applied Biosystems, Foster City, CA). PCR was conducted using Quick Taq HS Dye Mix (Toyobo, Osaka, Japan) and the primers listed in Table S1.

### 4.6. Sphingolipidomic Analysis

Total sphingolipids were prepared from lyophilized rice tissues and quantified by LC-MS/MS as described previously [25,48]. The lipid content was represented as nmol per g dry weight (DW). Free LCB composition was also analyzed using the same extract after NBD-F derivatization.

### 4.7. Measurement of Membrane Fluidity

Rice roots grown in Murashige and Skoog medium at 28 °C under a 12 h dark/12 h light (300 µmol/m^2^/s) cycle for 7 days were treated with 10 µM di-4-ANEPPDHQ (Molecular Probes) for 30 min. For observation under a confocal laser scanning microscope (FLUOVIEW FV3000, Olympus, Tokyo, Japan), fluorescence was excited with a 488-nm argon laser, and Lo and Ld images were obtained by measuring fluorescence at 500–550 nm and 650 to 750 nm, respectively (Figure S4). The fluorescence intensity of each image was measured by ImageJ (https://imagej.nih.gov/ij/), and a calibration of di-4-ANEPPDHQ was performed according to [13].

### 4.8. Aluminum Tolerance Assay

Aluminum tolerance in roots was evaluated by a hydroponic assay [49] using a hand-made floating container (Figure S5). Rice seedlings were grown on a plastic net floating on 0.5 mM CaCl_2_ (pH 4.5) at 28 °C under a 12 h dark/12 h light (300 µmol/m^2^/s) cycle for 4 days. For aluminum treatment, AlCl_3_ was added to the solution at a final concentration of 50 µM. Roots were photographed and their lengths measured before and 2 days after treatment. Relative root elongation was calculated as a ratio of [length elongated with Al^3+^]/[length elongated without Al^3+^].

## Supplementary Materials

The following are available online at https://www.mdpi.com/2223-7747/9/1/19/s1, Figure S1: A scheme of LCB unsaturation and distribution into glycosphingolipid classes. Figure S2: CRISPR/Cas9-mediated mutagenesis of *OsSLD*. Figure S3: CRISPR/Cas9-mediated mutagenesis of *OsDES*. Figure S4: Representative image of confocal microscopic analysis of plasma membrane fluidity. Figure S5: Representative image of hydroponically cultured rice for measurement of aluminum tolerance. Table S1: Primer sequences used in this study.
