# Supplementary material for: Plant-Unique cis/trans Isomerism of Long-Chain Base Unsaturation is Selectively Required for Aluminum Tolerance Resulting from Glucosylceramide-Dependent Plasma Membrane Fluidity
Source: Plants (Basel). 2019 Dec 23;9(1):19. doi: 10.3390/plants9010019 (PMC7020186; doi:10.3390/plants9010019)
Supplement: Supplementary file 1 [file plants-09-00019-s001.pdf]

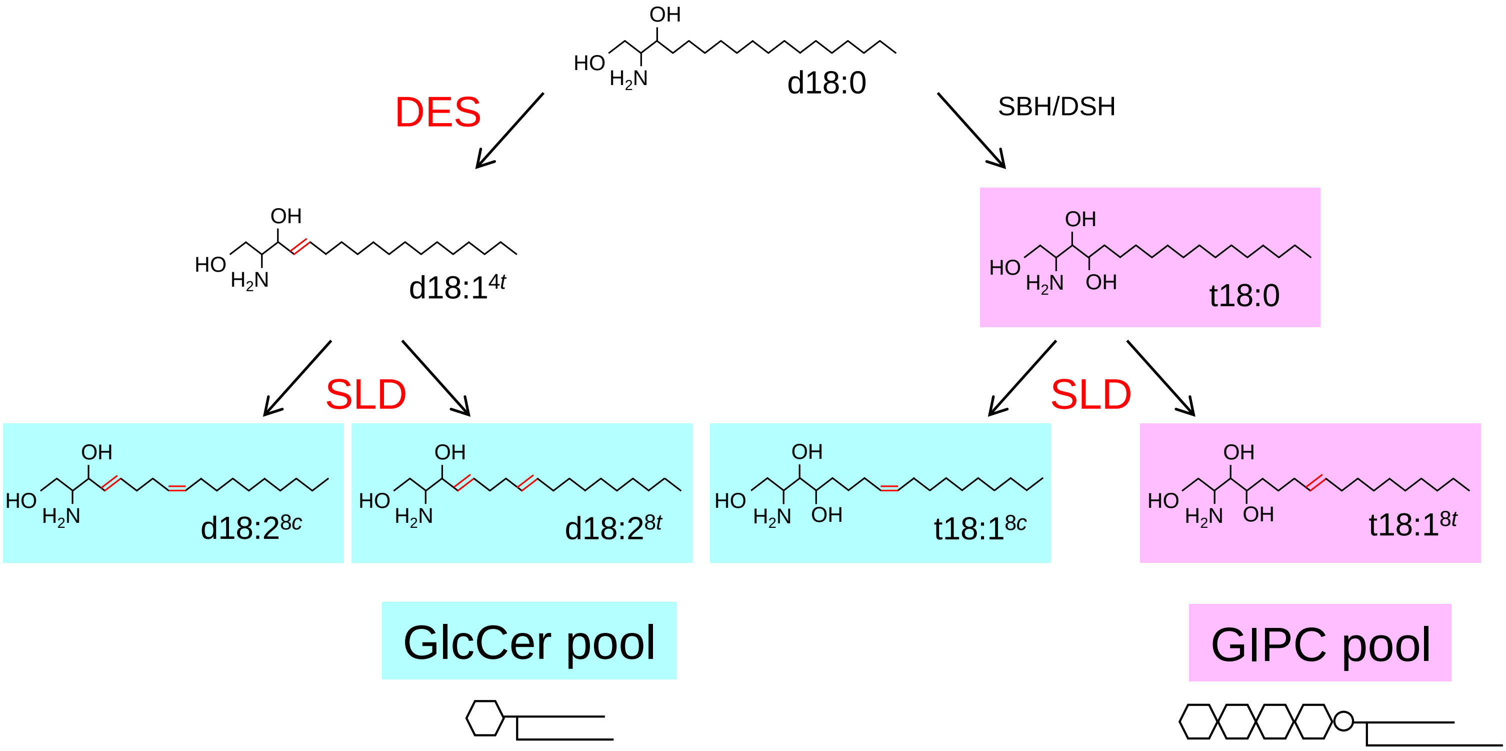

Supplementary Figure S1. A scheme of LCB unsaturation and distribution into glycosphingolipid classes.
Biological pathways of the LCB unsaturation and the selective incorporation into the GlcCer and GIPC pools that are estimated based on previous reports [3,5,25,35] are shown. The enzymes and the introduced double bonds that were modified in this study are indicated in red color. Note that the true substrates of the enzymes have not been identified (LCB or ceramide) but DES and SLD are estimated to use ceramide substrates, although this figure represents LCB structures. SBH, sphingoid base hydroxylase; DSH, dihydrosphingosine hydroxylase.

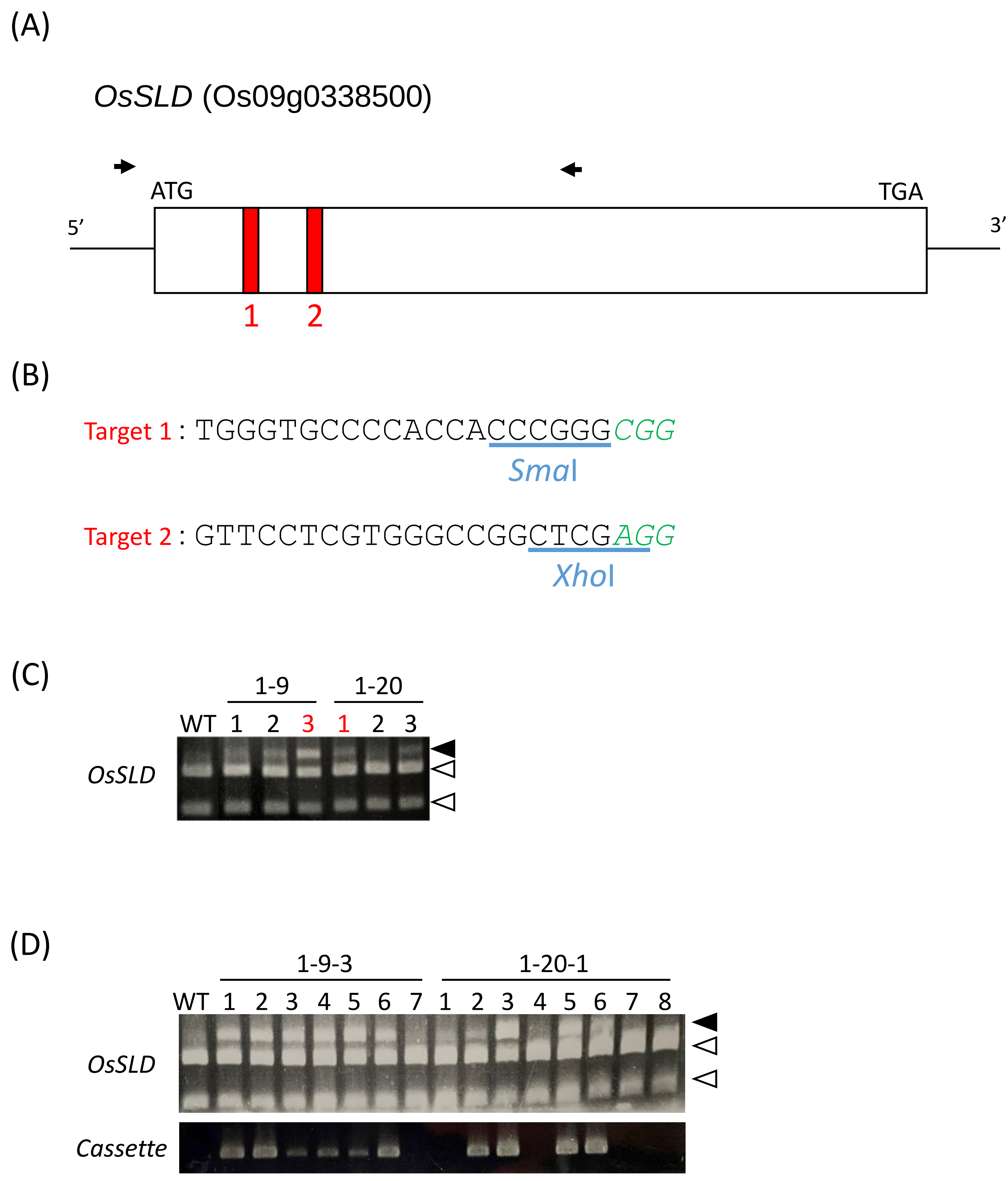

Supplementary Figure S2. CRISPR/Cas9-mediated mutagenesis of *OsSLD*.

(A) Location of target sites for CRISPR/Cas9-mediated mutagenesis of *OsSLD*. White box, coding sequence; lines, untranslated regions; red box, target sites. Arrows indicate primers used for genome PCR. (B) Nucleotide sequences and restriction sites of the two target sites. PAM (NGG) sequences are in italics. (C, D) PCR-restriction cutting genotyping of transgenic rice. Closed arrow, non-cut PCR products; open arrows, restriction fragments; WT, wild-type. (C) Two partially restriction-insensitive plants were isolated from the T1 generation of target 1, but nothing from target 2. (D) T2 seedlings of 1-9-3 and 1-20-1 progenies were further analyzed but no homozygous mutants were observed from 80 plants (partial data shown). In addition, all the plants with partially restriction-insensitive genome had the CRISPR/Cas9 cassette in their genome (lower panel), but plants with complete digested genome as WT did not. In addition, direct sequencing analysis of the undigested fragments indicated the co-presence of multiple mutations in the T2 generation plants. These results indicate that the detected genomic mutations occurred in the vegetative tissues rather than inherited from the T1 plants.

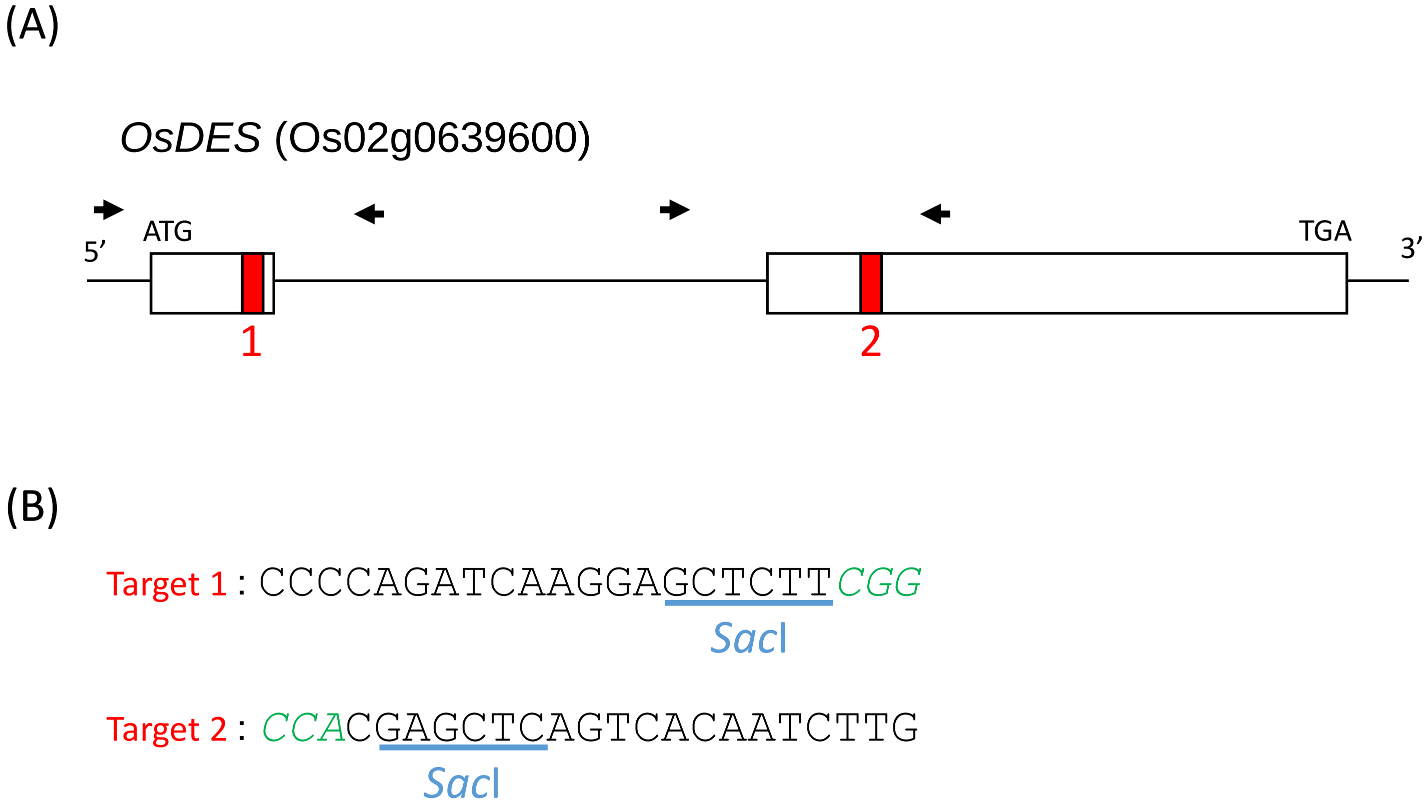

Supplementary Figure S3. CRISPR/Cas9-mediated mutagenesis of *OsDES*.
(A) Location of target sites for CRISPR/Cas9-mediated mutagenesis of *OsDES*. White box, coding sequence; solid lines, untranslated regions/intron; red box, target sites. Arrows indicate primers used for genome PCR. (B) Nucleotide sequences and restriction sites of the two target sites. PAM (NGG) sequences are in italics. Target 2 is reverse orientation.

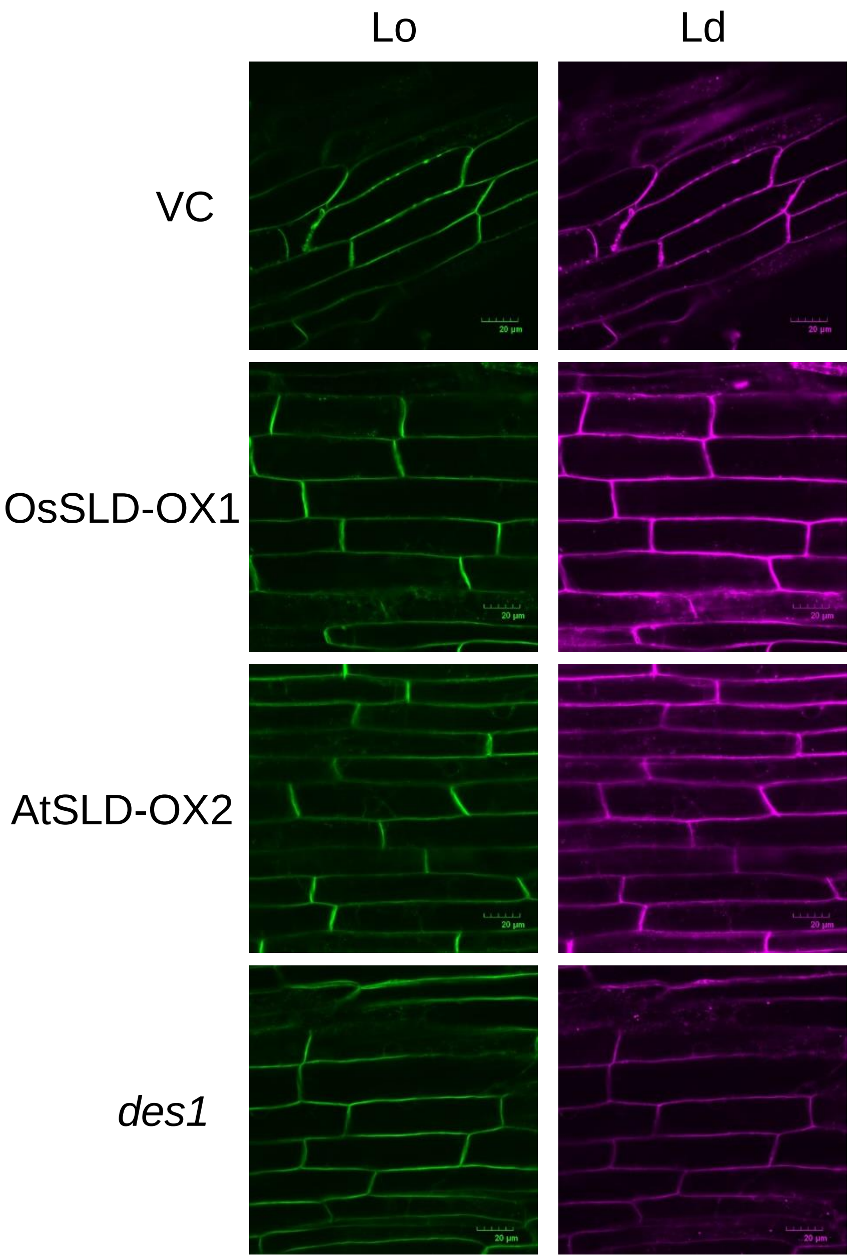

Supplementary Figure S4. Representative image of confocal microscopic analysis of plasma membrane fluidity.
Plasma membrane fluidity in rice roots was analyzed by confocal laser microscopy using di-4-ANEPPDHQ. The intensities of fluorescent emissions for Lo (green) and Ld (magenta) were used for normalization of their ratio as membrane fluidity.

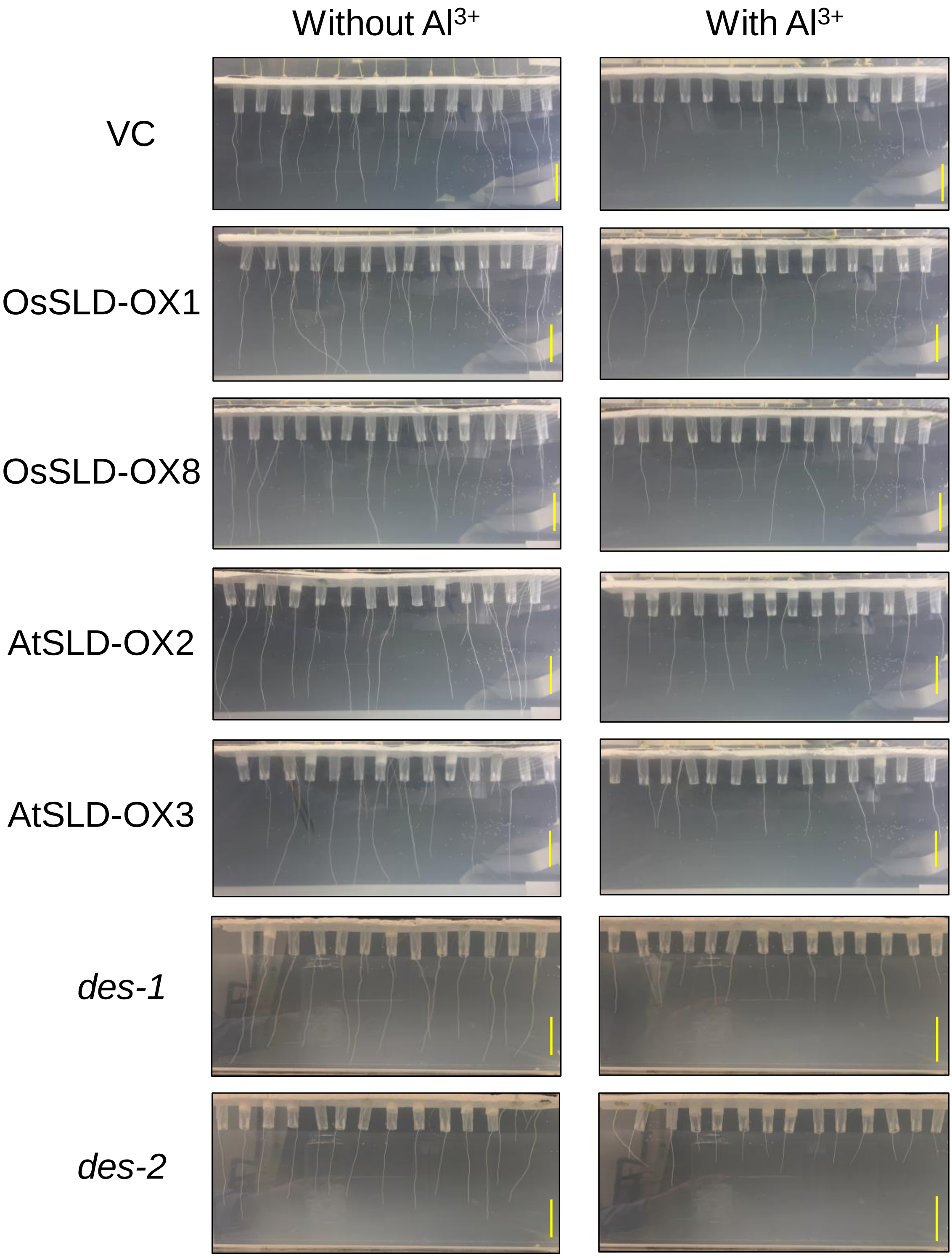

Supplementary Figure S5. Representative image of hydroponically cultured rice for measurement of aluminum tolerance. Rice seedlings were grown on a plastic net floating on solution with or without Al^{3+} and the elongated root length during 2-day-treatment was measured. Scale bars represent 3 cm.

Supplementary Table S1. Primer sequences used in this study.

Gene	Primer sequence	Experiment
OsSLD	F: TGCCTGAACCACTTCTCCTC	RT-PCR
	R: AGCAGCCCTCAATGTCTTCC	
	F: CTCACGTTCCCCCATCTCCGAATC	Genomic PCR
	R: CCGAACAGCTTGGAGGAGAC	
AtSLD1	F: ACGTTTCTCTTGCTCTTCTCCAAACG	RT-PCR
	R: GCTTGATAAGCTGCTGTCTTCAAAGTG	
OsEF1α	F: CTGCTGCTGCAACAAGATGG	RT-PCR
	R: CACGTCCTGAAGGGGAAGAC	
OsDES	F: CAGCCACCAAATCGACAAGAC	Genomic PCR for *des-1*
	R: AGTTTCAGCCGTAGTCAGTGCCTG	
	F: CTGGTTCTCCACTCTCGCTAACGC	Genomic PCR for *des-2*
	R: GGTTTTGGCTTGAGGAACAGC	
